# Interventions to Counter Health Misinformation Among Older People: Protocol for a Scoping Review

**DOI:** 10.2196/74138

**Published:** 2025-07-10

**Authors:** Maryline Vivion, Valérie Reid, Valérie Trottier, Frédéric Bergeron, Isabelle Savard, Emilie Dionne, André Tourigny

**Affiliations:** 1 Department of Social and Preventive Medicine Faculty of Medicine Laval University Quebec, QC Canada; 2 Centre Hospitalier Universitaire de Québec-Université Laval Research Center Quebec, QC Canada; 3 Laboratoire sur la Communication et le Numérique Montreal, QC Canada; 4 Direction des Services-Conseils de la Bibliothèque Laval University Quebec, QC Canada; 5 Education Department Université TÉLUQ Quebec, QC Canada; 6 VITAM – Research Centre for Sustainable Health Quebec, QC Canada; 7 Department of Sociology Faculty of Social Sciences Laval University Quebec, QC Canada; 8 Centre d'Excellence sur le Vieillissement de Québec Quebec, QC Canada

**Keywords:** misinformation, disinformation, older adults, intervention, health literacy

## Abstract

**Background:**

In contemporary society, misinformation and disinformation have emerged as significant challenges, impacting various aspects of public health and societal cohesion. Some authors argue that older adults are particularly vulnerable to the effects of misinformation due to potential digital health literacy challenges. A previous review identified pedagogical approaches most commonly adopted in interventions aiming to improve the digital literacy of older adults but did not specifically address digital health literacy.

**Objective:**

This scoping review protocol aims to explore digital health literacy interventions targeting health misinformation and designed specifically for older adults.

**Methods:**

Following the methodology outlined by Arksey and O'Malley and the PRISMA-ScR (Preferred Reporting Items for Systematic reviews and Meta-Analyses extension for Scoping Reviews) checklist, this protocol delineates a systematic approach encompassing 5 stages: identification of research questions, identification of relevant studies, selection of studies, data charting, and collation of findings. Our scoping review will include peer-reviewed literature on interventions targeting misinformation for older adults. Research will be conducted on the MEDLINE (Ovid), Embase (Elsevier), PsycINFO (Ovid), CINAHL, and Web of Science databases. Gray literature will also be surveyed by performing a Google search to identify interventions and tools employed by public or private organizations, institutes, groups, or agencies. The databases and gray literature will be searched to identify relevant publications. Two members of our team will independently select publications to include in the review by using the Covidence review software (Veritas Health Innovation). The publications included will specifically address our research questions, be peer-reviewed, evidence-based, and published from January 1, 2005, in full-text English or French version. Data will be extracted from the included publications to mainly chart the intervention’s objectives, types, target age groups, effectiveness, and risks reported. A thematic analysis will be conducted to categorize the study findings.

**Results:**

The funding for this project was provided in March 2024. The research questions were identified in January 2024. The databases and gray literature search strategies were developed in February 2024. The final selection of the publications; the charting, collating, and summarizing of data; along with the reporting of findings are planned for August to September 2025. The findings of this scoping review will be shared through publication in an open access journal and presentations scheduled between September and December 2025.

**Conclusions:**

This protocol will enable us to contribute to the advancement of knowledge in combating health misinformation among older adults. The results will also be utilized for the development of interventions targeting misinformation among older adults.

**International Registered Report Identifier (IRRID):**

PRR1-10.2196/74138

## Introduction

### Background

Misinformation and disinformation in health are significant issues because they undermine trust in public health authorities, scientists, and governments [1-4]. Health misinformation encompasses information that deviates from the established scientific consensus about a phenomenon [5]. Conversely, disinformation involves the intentional spread of inaccurate information with the aim of misleading or causing harm, while misinformation, although still incorrect, is shared without malicious intent [6]. In this protocol, the term “misinformation” will be used with regard to whether it is intended to deceive, as assessing intent can be challenging.

Health misinformation poses a threat to health, wellness, and also to democracies and social cohesion. Indeed, it can undermine trust in public health authorities, scientists, and governments; foster polarization; and fuel feelings of anxiety, fear, and depression [7,8]. Additionally, through its strong emotional appeal, it captures attention and distracts from pertinent information [9]. Health misinformation can also lead to misconceptions that may prompt potentially harmful actions [2,10]. Although its scope was highlighted by the recent COVID-19 pandemic, health misinformation is not a new phenomenon. Its negative consequences have previously been described for various public health topics such as vaccination, dietary regimes and disorders, drugs, new tobacco products, treatments for chronic diseases, and medication use [2,10-12], as well as in relation to climate change [1].

Whenever unreliable information is mentioned, the social and digital media are often called into question. Social media such as Facebook, X, Instagram, Reddit, or YouTube promote the dissemination of false information and fake news [13]. As the internet and social media become increasingly popular for finding health-related information [14], it is necessary to examine how people assess or evaluate the quality of information present on these platforms.

Older people (>60 years old) are often identified as more vulnerable to misinformation than other demographic groups due to their potential lack of necessary skills to retrieve and evaluate web-based information [15-17]. The concept of digital health literacy (eHealth literacy) is frequently mobilized in these studies. They encompass the ability to search, find, understand, and evaluate health information from electronic sources and to apply the acquired knowledge to address or resolve a health issue [18].

To address this issue, various interventions have been developed to enhance digital health literacy among older adults. Most interventions target the use of social media, online services, software, applications, and search engines and focus on older adults’ abilities to understand and evaluate web-based information [19]. A systematic review [19] identified the pedagogical approaches most commonly adopted in these interventions. The first type of approaches are formal pedagogies focused on teaching through fixed duration courses with predetermined content and objectives. These pedagogical designs often included peer learning support through collaborative tasks. The other type is pedagogies centered on the individual and their specific needs, which means they are based on the learning objectives of the participants, their experience, capabilities, or existing habits of using devices and technologies. Some studies identified in the systematic review observed an improvement in the abilities of their participants to use the internet, as well as an enhancement in their self-confidence and quality of life following the intervention, while other studies did not measure any changes. Although these interventions aimed to enhance literacy, they were not designed to specifically address digital health literacy as well as misinformation. This section can include background information such as theories, prior work, and hypotheses.

### Review Objectives

The objective of this scoping review is to describe digital health literacy interventions and tools that have been implemented to counter health misinformation and that are specifically targeting older adults, thereby providing a more nuanced exploration of this critical area.

## Methods

### Scoping Review Methodology

Given the breadth of topics covered by our research objective, a scoping review is particularly valuable to map the body of literature because it allows for an expansive exploration of the existing literature [20]. By systematically mapping the literature, scoping reviews provide a comprehensive overview of the available evidence, including diverse sources such as academic papers and gray literature [20]. This inclusive methodology will enable researchers to identify gaps, patterns, and key concepts within the literature landscape related to digital health interventions developed to counter health misinformation. To enhance the potential for impact, applicability, and co-production of evidence, an advisory committee, including public health professionals from the Ministry of Health and Social Services and other organizations as well as citizen partners, will be consulted throughout the different stages of the scoping review, including the refinement of the research questions, interpretation of the results, and the development and dissemination of findings.

### Protocol Design

This scoping review is based on the methodological design constructed by Arksey and O’Malley [21] and the enhanced version by Levac et al [20]. The PRISMA-ScR (Preferred Reporting Items for Systematic Reviews and Meta-Analyses extension for Scoping Reviews) checklist [22] was also used to develop this protocol (Multimedia Appendix 1). This design consisted of 5 stages.

#### Stage 1: Identification of the Research Question/Questions

The research question guiding our scoping review is as follows: what digital health literacy interventions, directed specifically toward older adults, have been deployed to address or counter health misinformation? By “interventions,’’ we are referring to various actions such as workshops, experiments, and the creation of toolkits.

#### Stage 2: Identification of Relevant Studies

With the collaboration of a specialized librarian (FB), we selected 5 databases relevant to our research question: MEDLINE (Ovid), Embase (Elsevier), PsycINFO (Ovid), CINAHL, and Web of Science. We identified 5 concepts to help us find relevant studies: older adults, digital health literacy, interventions, misinformation, and health. To make sure to target studies that align with the scope of our search criteria, we added the concept of “health” for PsycINFO and Web of Science, as PsycINFO is dedicated to psychology, and Web of Science encompasses multidisciplinary content. Including the term “health” in these searches helps to filter and focus on studies that are relevant to our specific area of interest in health research. We identified free-text keywords through the databases for each of the 5 concepts (Table 1) targeted by our research question.

Subsequently, we searched for corresponding controlled vocabulary employed by the MEDLINE, Embase, CINAHL, and PsycINFO databases by using their respective search tools, as this vocabulary is specific and variable between databases (Table 2). These 2 steps enabled us to establish the preliminary search strategies for each database (Multimedia Appendix 2).

**Table 1:** Concept-associated free-text keywords identified for search in databases.

Concept	Free-text keywords
Older adult	Older adult^a^, older people, old people, old person^a^, senior^a^, elder^a^, aged, geriatric^a^
Digital health literacy	Health literacy, medical literacy, health information literacy, eHealth literacy, digital health literacy^a^, technolog^a^, computer^a^, ICT, information and communications technolog^a^, digital illiteracy
Interventions	Interventions^a^, workshop^a^, training program^a^, course^a^, pedagogy, instruction^a^, skill^a^, learning, teaching, exposure^a^, tool^a^, resource^a^, app, apps, application^a^, device^a^, approach^a^, experiment^a^, experimental research^a^, prebunking, pre-emptive, debunking, reactive of fact-checking, inoculation, immunizing
Misinformation	Misinform^a^, disinform^a^, dis inform^a^, mis inform^a^, malinfor^a^, mal infor^a^, infodem^a^, infobesit^a^, rumor^a^, rumour^a^, hoax^a^, fallac^a^, conspirac^a^, myth, myths, gossip^a^, skeptic^a^, sceptic^a^, infoxication, veracity, polariz^a^, polaris^a^, controvers^a^, denial^a^, dessent^a^, contest^a^, deny, denier^a^, alarmism, contrarianism, reinformation, false belief^a^, falsehood^a^, trolls, post-truth, misconception, deception, ((inaccurate, false, fake, poor quality, low quality, misleading, distorted) AND (information^a^, news, communication^a^))
Health	Well-being, health, medical, public health, treatment^a^

^a^Various terminations are included.

**Table 2:** Concept-associated controlled vocabulary search terms identified to search in databases.

Concept	Controlled vocabulary search terms in the databases			
	MEDLINE	Embase	PsycINFO	CINAHL
Older adult	Aged, 80 and over	Aged, very elderly	Older adulthood, geriatric patients, aging	Aged, 80 and over
Digital health literacy	Health education, health literacy, internet, digital technology, computers, computer literacy	Health literacy, internet literacy, eHealth literacy, computer literacy, social media, information technology, communication technology, digital technology, computer, technology, health education	Digital literacy, health literacy, health promotion, technology, health information, health knowledge, health education, digital information, digital health resources, internet, computers, internet usage, computer usage, information and communication technology	Health literacy, computer literacy, social media, digital technology, digital health, health information, health education
Interventions	Computer user training, problem-based learning, simulation training, cognitive training, internet-based intervention, education, mentoring, teaching, learning, media exposure, software, mobile applications, immunization	Intervention study, web-based intervention, workshop, simulation training, learning, experiential learning, skill, mobile application, devices, human experiment, experimentation, immunization	Group intervention, intervention, training, ability, adult learning, learning, cooperative learning, teaching, exposure, computer applications, mobiles applications, experimental methods, prevention, immunization	Seminars and workshops, community programs, peer assistance programs, skill acquisition, learning, teaching, media exposure, information resources, mobile applications, experimental studies, quasi-experimental studies, immunization, immunization programs
Misinformation	Deception, disinformation, infodemic, denial	Misinformation, disinformation, propaganda, information overload, infodemic, conspiracy theory, denial, deception, public opinion, information dissemination	Misinformation, deception, information dissemination, conspiracy beliefs, conspiracy theories, gossip, skepticism, denial, false beliefs	Misinformation, disinformation, information avoidance, information explosion, medical mistrust, denial (psychology), propaganda, deception, scientific misconduct
Health	N/A^a^	N/A	Well-being, health, health attitudes, health behavior, public health, public health attitudes	N/A

^a^N/A: not applicable.

In the next phase, we will perform a gray literature overview (reports, theses, working papers, blogs, research data) to identify interventions and tools employed by public or private organizations, institutes, groups, or agencies that may not appear in scientific literature and that are related to our research concepts. Free-text keywords related to our concepts identified for the bibliographic database search strategies (Table 1) will be used to search through Google. The first 100 Google search results will be screened for relevant publications. Gray literature searches will be documented in a spreadsheet, which will table the following information for each search: date of search, terms or expressions used in search, number of results found, number of results retained, and comments. We will assess the relevance of the publications from the gray literature by using a simplified checklist itemizing authority, accuracy, coverage, objectivity, date, and significance. The searching through the databases and the gray literature will be performed by a research professional (VT).

#### Stage 3: Selection of Relevant and Reliable Studies

The selection of the publications will be piloted on a subset of potentially relevant sources and thereafter performed independently by 2 members of the research team: the principal investigator (MV) and a research professional (VT). Publications identified in stage 2 will be exported into the bibliographic management software EndNote and the Covidence platform (Veritas Health Innovation). Using Covidence, we will remove duplicates and select the publications relevant to our research based on our inclusion and exclusion criteria (Table 3) following Levac et al’s [20] methodological design. Team discussions will be held to address studies that present challenges for inclusion or exclusion. We will refine our research strategies if necessary.

**Table 3:** Criteria for including or excluding publications in the review.

Criteria category	Inclusion criteria	Exclusion criteria
Type of intervention	Interventions or tools to counter health misinformation among older people.	Interventions or tools to counter other type of misinformation (like political) among older people or other groups of populations. Interventions or tools to counter health misinformation among groups other than older people. Ideas for interventions or tools (that have not been performed).
Publication information (authors’ details and publication date)	Provides author’s name and publication date.	Does not provide publication date or author’s name.
Publication date	Published on or after January 1, 2005.	Published before January 1, 2005.
Text version	Available in full text version.	Not available in full text version.
Language of publication	Available in English or French.	Only published in language other than English or French.
Study type	Original paper, systematic literature review, meta-analysis, scoping literature review, textbook, book, thesis, or report.	Opinion piece, media article, written or video commentary, editorial, letter to the editor, dissertation, or conference proceeding.

Publications covering interventions or tools to counter health misinformation among older people will be included in the review in order to answer our research question. Publications covering interventions or tools to counter other types of misinformation (such as political misinformation within the context of elections) will be excluded, as our focus is solely on health misinformation. We will also exclude interventions dedicated to populations other than older adults. Publications suggesting ideas of interventions or tools (rather than describing interventions that have been implemented or tested) to counter health misinformation will be excluded. Publications not providing the author’s name and publication date will not be included, as they do not provide all the information required for data extraction. Material not available in full text version or only published in languages other than English or French will be excluded due to our inability to access full content. We chose the start date of January 1, 2005, as it marks the beginning of an increase in internet usage among older adults and interest in research literature regarding media literacy among this group of population [19,23]. Peer-reviewed original papers, systematic literature reviews, meta-analyses, and scoping literature reviews will be included, as the review process provides assurance of the validity of these evidence-based publications. Textbooks, books, theses, and reports will also be considered, as they are also typically reviewed. Quantitative, qualitative, and mixed methods studies will be included to cover the various aspects of our research concepts. Primary sources will be excluded if they have already been incorporated into a selected evidence synthesis, unless the data they contain are not reported in the evidence synthesis. Opinion pieces, media articles, written or video commentaries, editorials, letters to the editor, dissertations, or conference proceedings will not be considered, as they may not have been reviewed or be evidence-based. References cited by relevant publications selected from bibliographic databases and gray literature could also be examined for additional publications to include. Given the exploratory nature of scoping reviews, it is possible that research, screening, and selection of publications may lead to the identification of new research terms or concepts or new data sources [24]. The methodology may then need to be adapted accordingly. In this case, adjustments will be noted, justified, and reported through the final review.

#### Stage 4: Charting the Data From the Included Studies

To document the relevant information related to our research question, we will complete a data extraction form for each included publication. As this form is being iteratively developed for this specific review, a preliminary version, which will be subject to further adjustments, is presented here (Textbox 1).

**Preliminary data extraction form.**

Title of publication:

Type of publication:

Author’s name:

Year of publication:

Objective of the intervention:

Type of intervention:

Target age groups:

Effectiveness of the intervention:

Risks of the intervention:

Research limits:

Other:

As proposed by Pollock et al [25], the extraction form will be pilot tested independently by each of the 2 reviewers for 10 of each type of publication included in the review to ensure that the form is accurate and to make any necessary adjustments. Any discrepancies revealed by the tests will be discussed and resolved by consensus among the 2 reviewers, consulting a third team member if required. Revisions to the extraction form and justifications will be noted and reported in the final review.

Using the revised extraction form, the first reviewer will extract the data from each included publication. Data extracted from each publication will be screened by the second reviewer to validate accuracy and completeness [25]. If the extraction form needs to be further updated as part of an iterative process, these revisions and justifications will be also be noted and reported. Any inconsistencies arising occurring during data extraction will be discussed and settled by consensus between the 2 reviewers, consulting a third team member if necessary. Authors of the publications in this study could be contacted to retrieve and confirm data when needed.

The final version of the extraction form accompanied by the list and the definitions of the items extracted from publications will be made available in the final review. Data assumptions and simplifications will also be documented and presented in the final review.

#### Stage 5: Collating, Summarizing, and Reporting the Findings

An overview and description of all the included publications and extracted data will be presented in the final review. For each publication, the citations, characteristics, and relevant data will be reported in a tabular format. To collate those results, we will utilize qualitative methods such as thematic analysis to regroup data and identify key themes relevant to our research question and generate meaningful insights.

This scoping review will provide an overview on a broad range of interventions and tools that have been implemented to counter health misinformation and that are specifically targeting older adults. This scoping review will allow us to compare the different existing strategies, their advantages and disadvantages, their effectiveness, and their effects on older adults. Subsequently, the results will be summarized and reported in a clear and concise manner by using a narrative format or visual representations such as maps, diagrams, or tables, ensuring that important findings are accurately represented.

## Results

This project was funded in March 2024. The identification of the research questions was achieved by the research team in January 2024. The literature search strategies for the bibliographic databases were iteratively developed in February 2024 by FB, VR, VT, and MV. The gray literature search strategy was developed in July 2024 by VR and MV. The search through the bibliographic databases and the gray literature is planned for May to July 2025. The final selection of the publications; the charting, collating, and summarizing of data; as well as the reporting of findings is scheduled for August to September 2025. The findings of this scoping review will be disseminated through publication in an open access journal and presentations planned for September to December 2025. 

## Discussion

### Expected Outcomes

Our results will enhance the comprehensive overview of the literature on misinformation and contribute to advancing understanding in this field. The results will be valuable for guiding communication professionals dedicated to combating misinformation and effectively tailoring their message to the targeted population. Additionally, this scoping review marks a preliminary step in a project aimed at collaboratively developing interventions or tools with older adults to combat misinformation. The findings of this scoping review will serve as a foundational element in this endeavor.

### Limitations

This protocol for a scoping review has a few limitations. Language restrictions to English and French may result in the exclusion of relevant studies published in other languages. Since this scoping review aims to map the overall landscape of research on digital health literacy interventions and tools designed for older adults to counter health misinformation, approaches and definitions of misinformation used by the different included publications may vary substantially. This could make it challenging to extract data, collate, summarize, and collectively report the findings gathered from the different publications. Following delineated data extraction and analysis processes will facilitate the synthesis of the findings.

### Conclusions

This scoping review protocol will outline a comprehensive approach to investigate interventions aimed at addressing health misinformation among older adults. By recognizing the pervasive impact of misinformation on public health and social dynamics, this scoping review ensures a broad exploration of diverse literature sources, including gray literature, to capture a comprehensive understanding of the existing interventions. This scoping review aims to contribute to the advancement of knowledge in the field of misinformation, with implications for communication professionals and policy makers tasked with addressing this multifaceted challenge. Moreover, the protocol sets the stage for future collaborative efforts to develop targeted interventions tailored to the needs of older adults in combating misinformation. Thus, this scoping review not only serves as a critical exploration of existing literature but also as a foundational step toward meaningful intervention development and implementation.

## Appendix

Multimedia Appendix 1 PRISMA-ScR checklist.

Multimedia Appendix 2 Preliminary database search strategies.

Multimedia Appendix 3 Examples of prompts similar to those used for proofreading.

## Abbreviations

PRISMA-ScR: Preferred Reporting Items for Systematic reviews and Meta-Analyses extension for Scoping Reviews
