# Supplementary material for: Interventions to Counter Health Misinformation Among Older People: Protocol for a Scoping Review
Source: JMIR Res Protoc. 2025 Jul 10;14:e74138. doi: 10.2196/74138 (PMC12290425; doi:10.2196/74138)
Supplement: Multimedia Appendix 2 [file resprot_v14i1e74138_app2.docx]

**Preliminary DATABASES search strategies**

**MEDLINE:**

| 1 | Aged/ or exp "Aged, 80 and over"/ |
| --- | --- |
| 2 | (older adult? or older people or old people or old person? or senior? or elder* or aged or geriatric?).ti,ab,kf,kw |
| 3 | **1 OR 2** |
| 4 | exp Health Education/ or Health Literacy/ or Internet/ or Digital Technology/ or Computers/ or Computer Literacy/ |
| 5 | (health literacy or medical literacy or health information literacy or e-health literacy or digital health literacy or technolog* or computer? or ICT or information and communications technolog* or digital illiteracy).ti,ab,kf,kw |
| 6 | **4 OR 5** |
| 7 | Computer User Training/ or Problem-Based Learning/ or Simulation Training/ or Cognitive Training/ or Internet-Based Intervention/ or Education/ or Mentoring/ or Teaching/ or Learning/ or Media Exposure/ or Software/ or exp Mobile Applications/ or Immunization/ |
| 8 | (Intervention* or workshop? or training program? or course? or pedagogy or instruction* or skill? or learning or teaching or exposure? or tool* or resource? or app or apps or application? or device? or approach* or experiment* or experimental research* or prebunking or pre-emptive or debunking or reactive or fact-checking or inoculation or immunizing).ti,ab,kf,kw |
| 9 | **7 OR 8** |
| 10 | Deception/ or Disinformation/ or Infodemic/ or Denial/ |
| 11 | (misinform* or disinform* or dis inform* or mis inform* or malinfor* or mal infor* or infodem* or infobesit* or rumo?r* or hoax* or fallac* or conspirac* or myth or myths or gossip* or skeptic* or sceptic* or infoxication or veracity or polariz* or polaris* or controvers* or denial* or dessent* or contest* or deny or denier* or alarmism or contrarianism or reinformation or false belief* or falsehood? or trolls or post-truth or misconception or deception or ((inaccurate or false or fake or poor quality or low quality or misleading or distorted) adj3 (information* or news or communication*))).ti,ab,kf,kw |
| 12 | **10 OR 11** |
| 13 | **3 and (6 and 9) and 12** |

**Embase:**

| 1 | aged'/de or 'very elderly'/de |
| --- | --- |
| 2 | (older adult$ or older people or old people or old person$ or senior* or elder* or aged or geriatric$).ti,ab,kw |
| 3 | **1 or 2** |
| 4 | health literacy'/de or 'internet literacy'/de or 'ehealth literacy'/de or 'computer literacy'/de or 'social media'/de or 'information technology'/de or 'communication technology'/de or 'digital technology'/de or computer/de or technology/de or 'health education'/de |
| 5 | (health literacy or medical literacy or health information literacy or e-health literacy or digital health literacy or technolog* or computer$ or ICT or information and communications technolog* or digital illiteracy):ti,ab,kw |
| 6 | **4 or 5** |
| 7 | intervention study'/de or 'web-based intervention'/de or 'workshop'/de or 'simulation training'/de or 'learning'/de or 'experiential learning'/de or 'skill'/de or 'mobile application'/de or 'devices'/de or 'human experiment'/de or 'experimentation'/de or 'immunization'/de |
| 8 | (Intervention* or workshop$ or training program$ or course$ or pedagogy or instruction* or skill$ or learning or teaching or exposure$ or tool* or resource$ or app or apps or application* or device$ or approach* or experiment* or experimental research* or prebunking or pre-emptive or debunking or reactive or fact-checking or inoculation or immunizing):ti,ab,kw |
| 9 | **7 or 8** |
| 10 | misinformation'/de or 'disinformation'/de or 'propaganda'/de or 'information overload'/de or 'infodemic'/de or 'conspiracy theory'/de or 'denial'/de or 'deception'/de or 'public opinion'/de or 'information dissemination'/de |
| 11 | (misinform* or disinform*or dis inform* or mis inform* or malinfor* or mal infor* or infodem* or infobesit* or rumo?r* or hoax* or fallac* or conspirac* or myth or myths or gossip* or skeptic* or sceptic* or infoxication or veracity or polariz* or polaris* controvers* or denial* or dessent* or contest* or deny or denier* or alarmism or contrarianism or reinformation or false belief* or falsehood* or trolls or post-truth or misconception or deception or ((inaccurate or false or fake or poor quality or low quality or misleading or distorted) NEAR/3 (information* or news or communication*))):ti,ab,kw |
| 12 | **10 + 11** |
| 13 | **3 and (6 or 9) and 12** |

**PsycINFO:**

| 1 | older adulthood/ or geriatric patients/ or aging/ |
| --- | --- |
| 2 | (older adult? or older people or old people or old person* or senior* or elder* or aged or geriatric?).ti,ab,id |
| 3 | **1 or 2** |
| 4 | digital literacy/ or health literacy/ or health promotion/ or technology/ or health information/ or health knowledge/ or health education/ or digital information/ or digital health resources/ or internet/ or computers/ or internet usage/ or computer usage/ or information and communication technology/ |
| 5 | (health literacy or medical literacy or health information literacy or e-health literacy or digital health literacy or technolog* or computer? or ICT or information and communications technolog* or digital illiteracy).ti,ab,id |
| 6 | **4 or 5** |
| 7 | group intervention/ or intervention/ or training/ or ability/ or adult learning/ or learning/ or cooperative learning/ or teaching/ or exposure/ or computer applications/ or mobiles applications/ or experimental methods/ or prevention/ or immunization/ |
| 8 | (Intervention* or workshop? or training program* or course? or pedagogy or instruction* or skill* or learning or teaching or exposure? or tool* or resource? or app or apps or application* or device? or approach* or experiment* or experimental research* or prebunking or pre-emptive or debunking or reactive or fact-checking or inoculation or immunizing).ti,ab,id |
| 9 | **7 or 8** |
| 10 | misinformation/ or deception/ or information dissemination/ or conspiracy beliefs/ or conspiracy theories/ or gossip/ or skepticism/ or denial/ or false beliefs/ |
| 11 | (misinform* or disinform*or dis inform* or mis inform* or malinfor* or mal infor* or infodem* or infobesit* or rumo?r* or hoax* or fallac* or conspirac* or myth or myths or gossip* or skeptic* or sceptic* or infoxication or veracity or polariz* or polaris* or controvers* or denial* or dessent* or contest* or deny or denier* or alarmism or contrarianism or reinformation or false belief* or falsehood? or trolls or post-truth or misconception or deception or ((inaccurate or false or fake or poor quality or low quality or misleading or distorted) adj3 (information* or news or communication*))).ti,ab,id |
| 12 | **10 or 11** |
| 13 | well being/ or health/ or health attitudes/ or health behavior/ or public health/ or public health attitudes/ |
| 14 | (well-being or health or medical or public health or treatment*).ti,ab,id |
| 15 | **13 or 14** |
| 16 | **3 and (6 or 9) and 12 and 15** |

**CINAHL:**

| 1 | MH (Aged/ or ''Aged, 80 and Over +''/) |
| --- | --- |
| 2 | TI (older adult# or older people or old people or old person# or senior# or elder* or aged or geriatric#) OR AB (older adult# or older people or old people or old person# or senior# or elder* or aged or geriatric#) |
| 3 | **1 or 2** |
| 4 | MH (Health Literacy/ or Computer Literacy/ or Social Media/ or Digital Technology/ or Digital Health" or Health Information/ or Health Education/) |
| 5 | TI (health literacy or medical literacy or health information literacy or e-health literacy or digital health literacy or technolog* or computer# or ICT or information and communications technolog* or digital illiteracy) OR AB (health literacy or medical literacy or health information literacy or e-health literacy or digital health literacy or technolog* or computer# or ICT or information and communications technolog* or digital illiteracy) |
| 6 | **4 or 5** |
| 7 | MH (Seminars and Workshops/ or Community Programs/ or Peer Assistance Programs/ or Skill Acquisition/ or Learning/ or Teachning/ or Media Exposure/ or Information Resources/ or Mobile Applications/ or Experimental Studies/ or Quasi-Experimental Studies/ or Immunization/ or Immunization Programs/) |
| 8 | TI (Intervention# or workshop# or training program# or course# or pedagogy or instruction# or skil#* or learning or teaching or exposure# or tool# or toolkit# or resource# or app or apps or application# or device# or approach* or experiment# or experimental research* or prebunking or pre-emptive or debunking or reactive or fact-checking or inoculation or immunizing) OR AB (Intervention# or workshop# or training program# or course# or pedagogy or instruction# or skil#* or learning or teaching or exposure# or tool# or toolkit# or resource# or app or apps or application# or device# or approach* or experiment# or experimental research* or prebunking or pre-emptive or debunking or reactive or fact-checking or inoculation or immunizing) |
| 9 | **7 or 8** |
| 10 | MH (misinformation/ or disinformation/ or Information Avoidance/ or Information Explosion/ or Medical Mistrust/ or Denial (Psychology)/ or Propaganda/ or Deception/ or Scientific Misconduct/) |
| 11 | TI (misinform* or disinform* or dis inform* or mis inform* or malinfor* or mal infor* or infodem* or infobesit* or rumo?r# or hoax or hoaxes or fallac* or conspirac* or myth# or gossip# or skeptic* or sceptic* or infoxication or veracity or polariz* or polaris* or controvers* or denial* or dessent* or contest* or deny or denier* or alarmism or contrarianism or reinformation or false belief* or falsehood* or troll# or post-truth or misconception or deception or ((inaccurate or false or fake or poor quality or low quality or misleading or distorted) N3 (information# or news or communication#))) OR AB (misinform* or disinform* or dis inform* or mis inform* or malinfor* or mal infor* or infodem* or infobesit* or rumo?r# or hoax or hoaxes or fallac* or conspirac* or myth# or gossip# or skeptic* or sceptic* or infoxication or veracity or polariz* or polaris* or controvers* or denial* or dessent* or contest* or deny or denier* or alarmism or contrarianism or reinformation or false belief* or falsehood* or troll# or post-truth or misconception or deception or ((inaccurate or false or fake or poor quality or low quality or misleading or distorted) N3 (information# or news or communication#))) |
| 12 | **10 or 11** |
| 13 | **3 and (6 or 9) and 12** |

**Web of Science:**

| **1** | TI=(''older adult$'' or ''older people'' or ''old people'' or ''old person*'' or senior* or elder* or aged or geriatric$) |
| --- | --- |
| **2** | AB=(''older adult$'' or ''older people'' or ''old people'' or ''old person*'' or senior* or elder* or aged or geriatric$) |
| **3** | AK=(''older adult$'' or ''older people'' or ''old people'' or ''old person*'' or senior* or elder* or aged or geriatric$) |
| **4** | KP=(''older adult$'' or ''older people'' or ''old people'' or ''old person*'' or senior* or elder* or aged or geriatric$) |
| **5** | **1 or 2 or 3 or 4** |
| **6** | TI=(''health literacy'' or ''medical literacy'' or ''health information literacy'' or ''e-health literacy'' or ''digital health literacy'' or 'technolog* or computer$ or ICT or ''information and communications technolog*'' or ''digital illiteracy'') |
| **7** | AB=(''health literacy'' or ''medical literacy'' or ''health information literacy'' or ''e-health literacy'' or ''digital health literacy'' or 'technolog* or computer$ or ICT or ''information and communications technolog*'' or ''digital illiteracy'') |
| **8** | AK=(''health literacy'' or ''medical literacy'' or ''health information literacy'' or ''e-health literacy'' or ''digital health literacy'' or 'technolog* or computer$ or ICT or ''information and communications technolog*'' or ''digital illiteracy'') |
| **9** | KP=(''health literacy'' or ''medical literacy'' or ''health information literacy'' or ''e-health literacy'' or ''digital health literacy'' or 'technolog* or computer$ or ICT or ''information and communications technolog*'' or ''digital illiteracy'') |
| **10** | **6 or 7 or 8 or 9** |
| **11** | TI=(Intervention* or workshop$ or ''training program*'' or course* or pedagogy or instruction* or skill* or learning or teaching or exposure$ or tool* or resource$ or app or apps or application* or device$ or approach* or experiment* or ''experimental research*'' or prebunking or pre-emptive or debunking or reactive or fact-checking or inoculation or immunizing) |
| **12** | AB=(Intervention* or workshop$ or ''training program*'' or course* or pedagogy or instruction* or skill* or learning or teaching or exposure$ or tool* or resource$ or app or apps or application* or device$ or approach* or experiment* or ''experimental research*'' or prebunking or pre-emptive or debunking or reactive or fact-checking or inoculation or immunizing) |
| **13** | AK=(Intervention* or workshop$ or ''training program*'' or course* or pedagogy or instruction* or skill* or learning or teaching or exposure$ or tool* or resource$ or app or apps or application* or device$ or approach* or experiment* or ''experimental research*'' or prebunking or pre-emptive or debunking or reactive or fact-checking or inoculation or immunizing) |
| **14** | KP=(Intervention* or workshop$ or ''training program*'' or course* or pedagogy or instruction* or skill* or learning or teaching or exposure$ or tool* or resource$ or app or apps or application* or device* or approach* or experiment* or ''experimental research*'' or prebunking or pre-emptive or debunking or reactive or fact-checking or inoculation or immunizing) |
| **15** | **11 or 12 or 13 or 14** |
| **16** | TI=(misinform* or disinform*or dis inform* or mis inform* or malinfor* or mal infor* or infodem* or infobesit* or rumo?r* or hoax* or fallac* or conspirac* or myth or myths or gossip* or skeptic* or sceptic* or infoxication or veracity or polariz* or polaris* or controvers* or denial* or dessent* or contest* or deny or denier* or alarmism or contrarianism or reinformation or ''false belief*'' or falsehood* or trolls or post-truth or misconception or deception or ((inaccurate or false or fake or ''poor quality'' or ''low quality'' or misleading or distorted) NEAR/2 (information* or news or communication*))) |
| **17** | AB=(misinform* or disinform*or dis inform* or mis inform* or malinfor* or mal infor* or infodem* or infobesit* or rumo?r* or hoax* or fallac* or conspirac* or myth or myths or gossip* or skeptic* or sceptic* or infoxication or veracity or polariz* or polaris* or controvers* or denial* or dessent* or contest* or deny or denier* or alarmism or contrarianism or reinformation or ''false belief*'' or falsehood* or trolls or post-truth or misconception or deception or ((inaccurate or false or fake or ''poor quality'' or ''low quality'' or misleading or distorted) NEAR/2 (information* or news or communication*))) |
| **18** | AK=(misinform* or disinform*or dis inform* or mis inform* or malinfor* or mal infor* or infodem* or infobesit* or rumo?r* or hoax* or fallac* or conspirac* or myth or myths or gossip* or skeptic* or sceptic* or infoxication or veracity or polariz* or polaris* or controvers* or denial* or dessent* or contest* or deny or denier* or alarmism or contrarianism or reinformation or ''false belief*'' or falsehood* or trolls or post-truth or misconception or deception or ((inaccurate or false or fake or ''poor quality'' or ''low quality'' or misleading or distorted) NEAR/2 (information* or news or communication*))) |
| **19** | KP=(misinform* or disinform*or dis inform* or mis inform* or malinfor* or mal infor* or infodem* or infobesit* or rumo?r* or hoax* or fallac* or conspirac* or myth or myths or gossip* or skeptic* or sceptic* or infoxication or veracity or polariz* or polaris* or controvers* or denial* or dessent* or contest* or deny or denier* or alarmism or contrarianism or reinformation or ''false belief*'' or falsehood* or trolls or post-truth or misconception or deception or ((inaccurate or false or fake or ''poor quality'' or ''low quality'' or misleading or distorted) NEAR/2 (information* or news or communication*))) |
| **20** | **16 or 17 or 18 or 19** |
| **21** | TI=(well-being or health or medical or ''public health'' or treatment*) |
| **22** | AB=(well-being or health or medical or ''public health'' or treatment*) |
| **23** | AK=(well-being or health or medical or ''public health'' or treatment*) |
| **24** | KP=(well-being or health or medical or ''public health'' or treatment*) |
| **25** | **21 or 22 or 23 or 24** |
| **26** | **5 and (10 or 15) and 20 and 25** |
